# Immune checkpoints regulate acute myeloid leukemia stem cells

**DOI:** 10.1038/s41375-025-02566-x

**Published:** 2025-04-02

**Authors:** Chantal Reinhardt, Adrian F. Ochsenbein

**Affiliations:** 1 Tumor Immunology, Department for BioMedical Research (DBMR), University of Bern, Bern, 3008 Switzerland; 2 Department of Medical Oncology, Inselspital, Bern University Hospital, University of Bern, Bern, 3010 Switzerland; 3 Present Address: Department of Medicine Solna, Karolinska Institutet, and Center for Molecular Medicine, Karolinska University Hospital, SE-141 86 Stockholm, Sweden

**Keywords:** Cancer stem cells, Acute myeloid leukaemia, Cancer microenvironment, Tumour immunology, Immunotherapy

## Abstract

Acute myeloid leukemia stem cells (LSCs) express major histocompatibility complex (MHC) class I and II and many different immune checkpoint ligands and receptors, in which respect they resemble professional antigen-presenting cells. In addition, LSCs reside in the bone marrow (BM), a primary and secondary lymphoid organ, surrounded by immune cells. The function of these immune checkpoints (ICs) in the regulation of an anti-tumor immune response is well studied and IC inhibitors (ICIs) became a standard of care in many solid tumors. However, ICIs have very limited efficacy in AML. Nevertheless, the expression especially of immune activating ligands and receptors on LSCs is somewhat unexpected, since these cells have to evade protective immunity. Many ICs have been shown to mediate direct signaling in AML blasts and LSCs and thereby regulate self-renewal, differentiation and expansion of leukemic cells. Thus, the expression of ICs on the cell surface or their soluble forms often correlate with worse survival. In this review we summarize recent data on selected ICs of the immunoglobulin superfamily (IgSF) and the tumor necrosis factor receptor superfamily (TNFRSF) that have a documented role in the regulation of LSCs, independent of their immune regulatory role, and might become novel therapeutic targets.

## Introduction

Acute myeloid leukemia (AML) is a heterogenous group of hematological malignancies, characterized by rapid proliferation and accumulation of immature myeloid cells in the bone marrow (BM) and peripheral blood. Ultimately, this impairs normal hematopoiesis and leads to bone marrow failure [1]. Similar to normal hematopoietic stem cells (HSCs), a subpopulation of leukemic cells, termed leukemic stem cells (LSCs), is capable of reconstituting leukemia in recipient mice upon transplantation. LSCs have the potential of self-renewal and differentiation to leukemia blasts. In addition, they possess mechanisms that mediate resistance to chemotherapy and immunosurveillance. Therefore, LSCs are responsible for leukemia initiation, maintenance, and relapse [1]. LSCs share the BM microenvironment with healthy HSCs, and interactions with BM resident cells lead to a permissive environment for leukemogenesis [1].

### Interactions of LSCs with their niche

HSCs are regulated by cell-intrinsic factors (e.g., active signaling pathways, epigenetic cell state), as well as by environmental cues (e.g., soluble factors, cellular interactions, biophysical cues) [1, 2]. The specialized microenvironment of the HSC, called “niche”, contributes such cell-extrinsic factors regulating HSC characteristics, and is essential for HSC self-renewal, proliferation, and inhibition of differentiation [1]. Similarly, proliferation and the balance between self-renewal and differentiation of LSCs is regulated by niche cells and a dysfunction of these cells can contribute to leukemogenesis [3].

Both the HSC and LSC niche consist of different cell types, including mesenchymal stem cells, osteoblasts, adipocytes, perivascular and endothelial cells, as well as extracellular matrix components [3, 4]. In addition, the BM is a primary and a secondary lymphoid organ, and various mature immune cells, such as T-, B-, plasma- and dendritic cells, neutrophils and macrophages are present in close proximity to HSCs and LSCs [4, 5]. In leukemia, the LSC niche has been turned into a pro-inflammatory environment supporting LSC survival and proliferation. Several pro-inflammatory cytokines, produced by immune or non-immune cells of the niche or by leukemic cells themselves, have been shown to promote leukemia cell survival and proliferation, such as IFNγ, TNFα, IL-1β, GM-CSF, IL-3 or IL-6. Vice versa, anti-inflammatory cytokines such as IL-1Rα, TGF-β and IL-10 have an inhibitory effect [6–9]. Immune cells have been shown to actively contribute to the regulation of both HSCs and LSCs [4, 5, 8, 9]. For example, a CD150^high^-subpopulation of niche-associated regulatory T-cells (Tregs) has been found to secrete adenosine, which via binding to A2AR on HSCs maintained their quiescence and protected them from oxidative stress [10]. LSCs and leukemic bulk cells express both MHC class I and class II molecules on which they present immunogenic peptides to CD8^+^ and CD4^+^ T-cells, respectively [11]. In addition, LSCs express a variety of immunomodulatory proteins, and therefore resemble professional antigen-presenting cells in many aspects [1]. Thus, LSCs crucially regulate the immune response in the BM [12]. Interestingly, many of these immune checkpoint molecules additionally regulate the function of LSCs directly (Tables 1 and 2) – an unexpected recently discovered role on which this review will focus.

**Table 1:** Expression and prognostic relevance of ICs of the IgSF in AML.

Immune Checkpoint (gene name)	Alternative names	Subfamily	Expression in AML	Correlation with clinical parameters	Refs
PD-1 (*PDCD1*)	CD279	CD28-subfamily of IgSF	mRNA and protein increased in CD34^+^ BM cells of AML patients compared to HC	PD-1 on AML blasts correlates with longer DFS	[29–32]
PD-L1 (*PDCD1LG1*)	CD274, B7-H1	B7-subfamily of IgSF	PD-L1 expressed in 20% of AML patients, elevated levels on LSC compared to bulk cells	PD-L1 on AML blasts is associated with poor OS, relapse, and cytogenetic mutations of the adverse risk group	[32, 37–44, 50]
CTLA-4 (*CTLA4*)	CD152	CD28-subfamily of IgSF	mRNA increased in PBMCs of AML compared to HC, surface protein detectable in >50% and cytoplasmic protein in 100% of AML patients	High CTLA-4 mRNA in AML TCGA data correlates with poor OS. No correlation with chemoresistance	[44, 52, 57, 58]
CD86 (*CD86*)	B7-2	B7-subfamily of IgSF	50% of patients express CD86 on CD45^+^CD34^+^c-Kit^+^ cells	High CD86 mRNA or protein correlates with poor OS	[57, 62–64, 68]
CD276 (*CD276*)	B7-H3	B7-subfamily of IgSF	Absent in HC, while detected on CD45^+^CD34^+^c-Kit^+^ cells in 30% of AML patients	CD276 mRNA in PBMC and 2Ig isoform expression correlate with worse OS, however patients with CD276^+^ AML show improved event-free survival	[64, 70, 73–76]
VTCN1 (*VTCN1*)	B7-H4, B7S1, B7x	B7-subfamily of IgSF	Upregulated in human cord blood CD34^+^ cells transduced with MLL-AF9	VTCN1 mRNA correlates with improved OS	[81, 82]
VISTA (*VSIR*)	B7-H5, PD-1H, Dies1	CD28-subfamily of IgSF	Elevated mRNA levels, and protein is detectable in most AML patients. Higher VISTA expression in CD34^+^ than CD34^-^ BM cells of AML patients	VISTA mRNA levels are a negative prognostic factor in AML	[83, 86, 88]
CD28H (*TMIGD2*)	IGPR-1	CD28-subfamily of IgSF	Detected on CD34^+^ cells of >70% of AML patients, and protein levels are higher in CD34^+^ cells from AML than HC. Elevated CD28H positivity in LSC-enriched subpopulations	CD28H mRNA correlates with worse OS in TCGA dataset	[91]
TIM-3 (*HAVCR2*)	CD366, TIMD-3	TIM-subfamily of IgSF	Detected on LSCs of >75% of AML patients, increased expression in LSCs compared to blasts	TIM-3 expression on blasts correlates with poor prognostic markers and worse OS	[87, 93, 97–100]
LAIR-1 (*LAIR1*)	CD305	LAIR-subfamily of IgSF	Uupregulated in AML patient samples and cell lines	–	[103–106]

Summary of the expression ICs of the IgSF and their correlation with clinical outcome.

*OS* overall survival, *DFS* disease-free survival, *HC* healthy controls, *KO* knockout, *KD* knockdown.

**Table 2:** Expression and prognostic relevance of ICs of the TNFRSF/TNFSF in AML.

Immune checkpoint (gene name)	Alternative names	Subfamily	Expression in AML	Correlation with clinical parameters	Refs
TNFRSF3 (*LTBR*)	TNFBR	TNFRSF	Expressed on AML LSCs	Low LTβR or LIGHT levels correlate with improved OS	[110, 111]
CD27 (*CD27*) and CD70 (*CD70*)	TNFRSF7 and TNFSF7, CD27L	TNFRSF resp. TNFSF	CD27 and CD70 detected on most AML patient samples while absent on HC	Soluble CD27 correlates with worse prognosis	[119]
TNFRSF17 (*TNFRSF17*) and TNFSF13 (*TNFSF13*) and TNFSF13B (*TNFSF13B*)	BCMA, CD269 and APRIL, CD256, TALL2, TRDL1 and BAFF, CD257, TALL1, THANK	TNFRSF resp. TNFSF resp. TNFSF	BCMA^+^ subpopulations present in some AML patients	High frequency of BCMA^+^ blasts associated with CR after induction chemotherapy. High frequency of APRIL^+^ and BAFF^+^ blasts associated with NR after induction chemotherapy	[127–129]

Summary of the expression of ICs of the TNFRSF/TNFSF and their correlation with clinical outcome.

*HC* healthy controls, *CR* complete remission, *NR* no response.

### Immune checkpoint molecules (IC) and IC signaling

The term immune checkpoint molecules (IC) comprises a broad variety of cell surface proteins and glycan structures, including both receptors and ligands, which exert co-stimulatory or co-inhibitory effects on immune responses [13]. Although ICs can be expressed on a broad variety of cells, including tumor cells, their function is best documented in the regulation of B- and T-cell activation [13, 14]. Most ICs belong to two major superfamilies: the immunoglobulin superfamily (IgSF) and the tumor necrosis factor receptor superfamily (TNFRSF) with their respective ligands (TNFSF) [14].

**Fig. 1:**
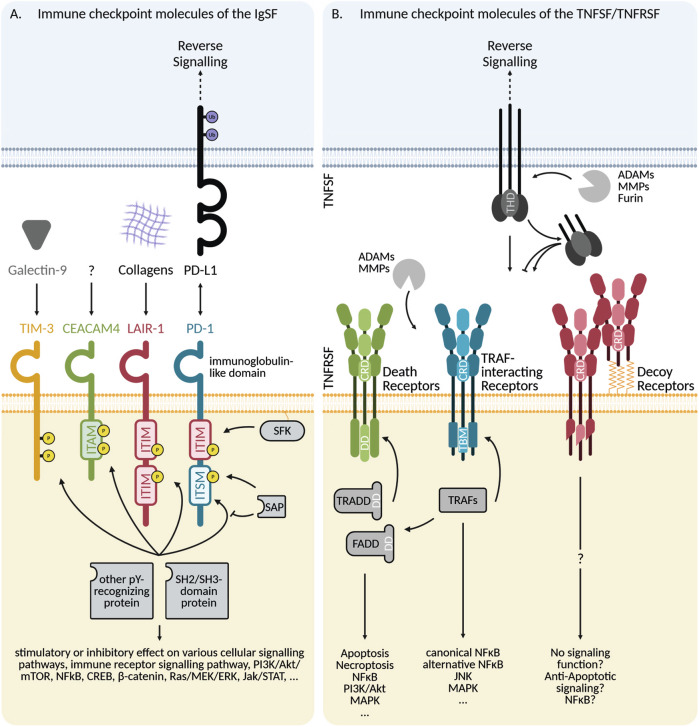
Signaling mechanisms of immune checkpoint molecules. **A** Signaling mechanisms of ICs of the IgSF. Most IgSF ICs signal via cytoplasmic ITRMs or other tyrosine-containing motifs. ITRMs can be activating (ITAM), inhibitory (ITIM), or switching between both (ITSM), which are generally associated with stimulating, dampening, or context-dependent influence on immune responses, respectively. Ligand binding induces tyrosine phosphorylation by SFKs, which enables the recruitment of SH2- or SH3-domain containing protein that delivers activating or inhibitory downstream signals. If a SH2-containing adaptor protein of the SAP-family is expressed, it can bind to a phosphorylated ITSM, preventing the binding of other SH2-/SH3-containing proteins and switching from inhibitory to stimulatory signaling by recruiting a different set of adaptor proteins. Several ICs of the IgSF signal via other signaling motifs than ITRMs. Certain ligand ICs of the IgSF engage in reverse signaling, either through ITRMs or other signaling motifs often involving phosphorylation or ubiquitination. **B** Signaling mechanisms of ICs of the TNFRSF and TNFSF. Generally, TNFRSF ICs are activated by TNFSF ligands, typically homotrimers with a THD. Most TNFSFs are synthesized as transmembrane proteins, but can be proteolytically cleaved into soluble variants. Some TNFRSFs are activated by both the membrane-bound and soluble variants of the ligand, while in others no signaling is induced even though there is an interaction between the receptor and the soluble ligand. TNFRSF contain at least one CRD, and are divided into three groups based on their intracellular composition: death receptors (DRs), TRAF-interacting receptors, and decoy receptors (DcRs). DRs use DDs to recruit DD-containing adaptor proteins such as FADD or TRADD, triggering cell death and other signaling pathways. TRAF-interacting receptors recruit members of the TRAF family (TRAF1-7) through TRAF-binding motifs and induce pathways such as NFκB, MAPK, or JNK. Some TRAF-members are also involved in DR-signaling through interaction with FADD or TRADD. DcRs do not possess complete intracellular signaling domains or are secreted proteins, thus they mainly act via ligand sequestering. Nevertheless, direct influence on some signaling pathways has been described. Non-DcR TNFRSFs can also exist as soluble forms through proteolytical cleavage of their membrane bound forms, often after signaling has been induced. Some members of the TNFSF mediate reverse signaling and influence pathways such as NFκB, MAPK, or PI3K. IC Immune Checkpoint, IgSF immunoglobulin superfamily, ITRM immunoreceptor tyrosine-based regulatory motif, ITAM immunoreceptor tyrosine-based activating motif, ITIM immunoreceptor tyrosine-based inhibitory motif, ITSM immunoreceptor tyrosine-based switch motif, SAP SLAM-associated protein, SFK Src Family Kinases, ITK Interleukin-3-inducible T-cell Kinase, P phosphorylated amino acid residue, Ub ubiquitinated site, TNFSF tumor necrosis factor superfamily, TNFRSF tumor necrosis factor receptor superfamily, ADAM a disintegrin and metalloprotease, MMP matrix-metalloprotease, THD TNF homology domain, CRD cysteine-rich domain, DD death domain, FADD Fas-associated death domain, TRADD TNF receptor associated death domain, TBM TRAF-binding motif, TRAF TNF-Receptor Associated Factor. Created in BioRender.com.

Encompassing over 700 cell surface and soluble proteins, the IgSF is a large and functionally diverse group of proteins, unified by the presence of at least one immunoglobulin domain. Due to structural stability despite changes in its amino acid structure, the immunoglobulin domain is well suited for ligand binding, which explains why many IgSF proteins are involved in cell recognition, adhesion, developmental processes, and both innate and adaptive immune responses [15]. Currently, an immunomodulatory role has been described for more than 30 members of the IgSF, establishing them as IC molecules [14]. ICs of the IgSF can be further classified into functional subfamilies using the Brotherhood algorithm, which detects shared evolutionary related sequences [16]. This classification aligns well with historically defined groups based on both structure and function of the IC molecules, and results in subfamilies of IC of the IgSF such as the CD28, B7, or SLAM families, and many more [14, 16]. However, even when IC molecules have similar types, numbers and organization of Ig- and other domains, the intracellular signal transduction domain may vary substantially, leading to functionally distinct effects. CD28 and CTLA-4 for example both belong to the CD28 IgSF-subfamily and share a high sequence identity, yet they have opposing effects on T-cell activation: CD28 is the most potent and essential co-stimulatory signal for T-cells, while CTLA-4 opposes CD28 function by binding to the same ligand with a higher affinity and recruiting phosphatases to its cytoplasmic tail which counteract CD28 signaling [14]. Signaling of ICs of the IgSF (Fig. 1A) often encompasses intracellular immunoreceptor tyrosine-based regulatory motifs (ITRMs), which can either be activating (ITAM), inhibitory (ITIM) or both depending on cellular context (“switch”, ITSM) [17]. Upon ligand binding, ITRM and other tyrosine-containing motifs are phosphorylated by Src-family kinases, which is then recognized by SH2- or SH3-domain containing proteins [18]. In the case of co-stimulatory proteins such as CD28, these SH2-/SH3-containing proteins are often kinases which either enhance the activating immune cell signaling or engage additional pathways (e.g., PI3K binding to phosphotyrosine on CD28). In contrast, co-inhibitory receptors often recruit SH2-/SH3-containing phosphatases which counteract the activating signaling cascades or suppress the cellular activation in independent ways (e.g. PD-1 inhibiting RAS and ERK) [14]. Several signal transducers involved in these pathways, e.g. Src-family kinases, are also involved in signaling pathways of cytokines, growth factors, regulators of apoptosis, and many others [18].

The second IC superfamily comprises members of the TNFRSF or TNFSF, which includes 29 receptors and 19 ligands involved in diverse biological processes such as proliferation, differentiation, viability and modulation of the immune system (Fig. 1B) [19]. Members of the TNFSF are characterized by the presence of a TNF homology domain (THD), which mediates the ligand trimerization necessary for functionality [19]. Similarly, members of the TNFRSF contain at least one conserved cysteine-rich domain (CRD) orchestrating receptor trimerization and ligand interaction [19]. Although TNFSF-members are synthesized in a membrane-bound form, most of them can be proteolytically cleaved, thus generating a soluble variant. Importantly, not all soluble forms of TNFSF-molecules are capable of eliciting signaling in their corresponding receptors even though a high-affinity interaction occurs [19]. TNFRSF-members can be classified into three groups based on their intracellular interaction partners: death receptors (DRs), TRAF-interacting receptors, and decoy receptors (DcRs) [19]. DRs contain a death-domain in their cytoplasmic tail and interact with death-domain-binding proteins. However, despite their name, it is now recognized that DRs can stimulate cellular pathways other than induction of apoptosis, such as activation of NFκB and MAP kinases [19, 20]. As the name suggests, TRAF-interacting receptors contain a short motif allowing for recruitment of TNF-receptor associated factors (TRAFs), a family of proteins functioning both as ubiquitin ligases and scaffold proteins that is involved in signaling transduction of Toll-like receptors, NOD-like receptors, and some cytokine receptor families [21]. Finally, DcRs either lack a complete intracellular signaling domain or are secreted proteins. Thus, they are generally considered unable to transduce a signal but act via competitive binding of TNFSF ligands. Additionally, the cleaved forms of other TNFRSF-members can function as decoy receptors [19].

Overall, IC signaling is complex due to several aspects: (I) many receptors share identical ligands, while many ligands can bind to several receptors, (II) effects of signaling may depend on the cellular context, where a receptor-ligand-interaction can elicit a different response depending on the cellular context, (III) for many members of the IC family, upon receptor-ligand-interaction a signal is not only transmitted in the cell expressing the receptor (forward signaling), but also in the cell expressing the ligand (reverse signaling), (IV) in many cases ligands and receptors can be proteolytically cleaved and lead to soluble forms, which can have, depending on the molecule, the same, no, or even the opposite effect [19].

### Treatment with immune checkpoint inhibitors in AML

**Fig. 2:**
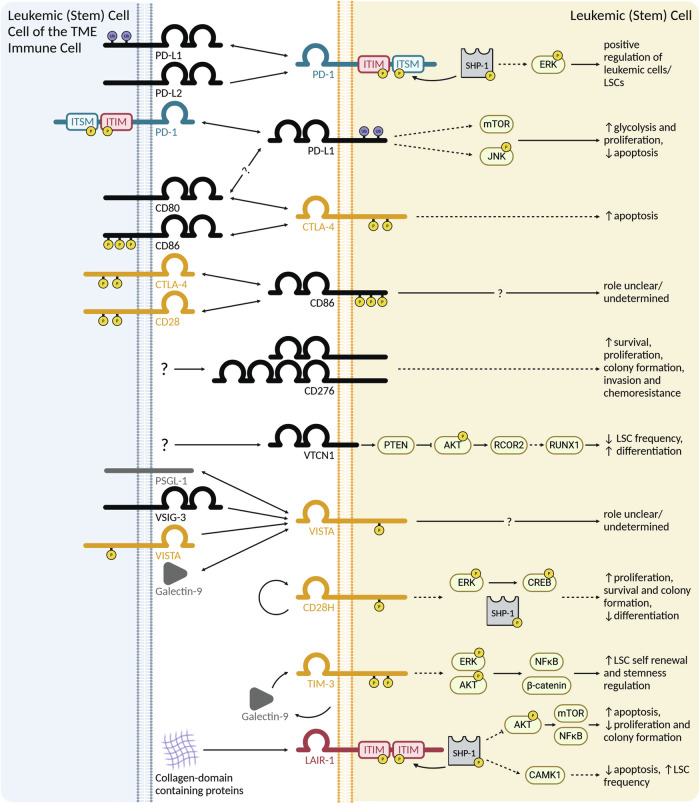
ICs of the IgSF regulating leukemia and LSC function. Overview of ICs of the IgSF with documented effects on AML cells and LSCs. TME tumor microenvironment, ITIM immunoreceptor tyrosine-based inhibitory motif, ITSM immunoreceptor tyrosine-based switch motif, LSC leukemic stem cell, P phosphorylated amino acid residue, Ub ubiquitinated site. Created in BioRender.com.

**Fig. 3:**
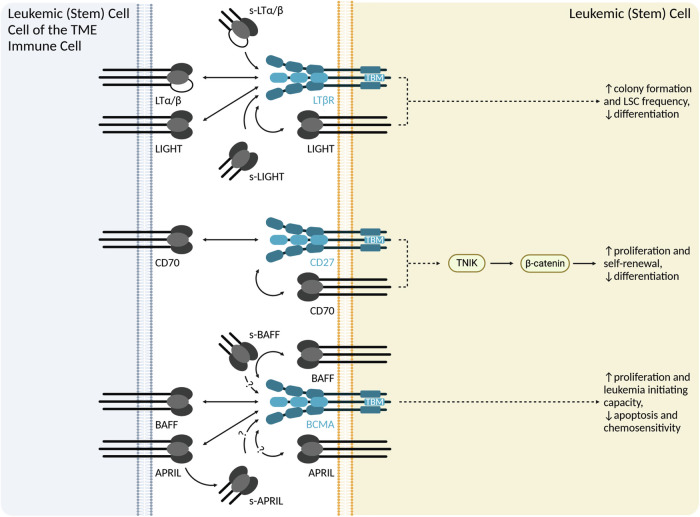
ICs of the TNFSF/TNFRSF regulating leukemia and LSC function. Overview of ICs of the TNFSF/TNFRSF with documented effects on AML cells and LSCs. TME tumor microenvironment, TBM TRAF-binding motif, LSC leukemic stem cell. Created in BioRender.com.

AML is an immunogenic disease and immune cells can target and eliminate leukemic blasts and LSCs. This is best documented after allogeneic hematopoietic stem cell transplantation (alloHSCT), where T-cells primarily directed against minor histocompatibility antigens are responsible for the graft-versus-leukemia response and eliminate leukemic cells [22]. However, leukemia-specific CD8^+^ and CD4^+^ T-cells have also been described in the autologous setting [22]. Thus, there is a strong rationale to re-enforce T-cell activity in AML by using ICI, and several clinical trials have been conducted or are ongoing (Table 3). Unfortunately, ICIs have shown modest activity in AML compared to solid tumors [23]. Efforts to improve their efficacy include their combination with chemotherapy or hypomethylating agents (HMA). Indeed, HMAs have been shown to upregulate immune checkpoints on both leukemic and immune cells, as well as tumor antigen presentation, providing the rationale to combine ICIs with HMAs [23]. In relapsed/refractory AML, the PD-1 blocking antibodies pembrolizumab and nivolumab in combination with the HMA azacitidine induced response rates of approximately 30% that were of short duration [24]. The addition of the PD-L1 blocking antibody durvalumab to azacitidine did not improve outcome [25]. Better efficacy of ICIs is observed in post-alloHSCT relapse patients [24]. The limited efficacy of ICIs in AML may be a consequence of a low mutational burden in AML, resulting in low-antigenicity of the malignant cells. Additionally, T-cell dysfunction may be much more severe in AML than in solid tumors. Indeed, we found that important genes involved in CD8^+^ T-cell activation, differentiation, and function are down-regulated in AML due to pathologic epigenetic alterations mediated via histone deacetylation [9]. As a consequence, CD8^+^ T-cells in AML may be dysfunctional not primarily due to the expression of inhibitory molecules, but due to the epigenetic silencing of immune activating receptors [26]. Although antibodies that block immune-inhibitory ICs in AML have not resulted in substantial immune activation and leukemia control, targeting ICs that are specifically expressed on LSCs and lead to their activation might be a promising novel approach to treating AML. This review focuses on this specific role of ICs of the IgSF (Fig. 2) and the TNFRSF/TNFSF (Fig. 3), independent of their immune regulatory function.

**Table 3:** Clinical trials employing antibodies targeting ICs in AML.

Target	Drug name	Strategy	Disease status (AML only)	Clinical phases	(Estimated) Enrollment	Start date	(Estimated) Primary completion	(Estimated) Study completion	Status	Response/survival if completed	Trial identifier
PD-1	Nivolumab	Nivolumab + idarubicin + cytarabine	De novo and R/R AML (R/R only in Phase I)	I/II	44	07/2015	05/2020	05/2020	Completed	CR/CRi 80%, median OS 18.5 months	NCT02464657
PD-1	Nivolumab	Nivolumab	AML in remission at high risk for relapse	II	15	10/2015	08/2023	08/2023	Completed	CR 71%, OS 67% at 18 months	NCT02532231
PD-1	Nivolumab	Nivolumab + azacitidine or azacitidine	De novo AML in elderly patients	II/III	49	02/2018	08/2023	06/2024	Completed	CR/CRi 20% vs. 24, median OS 5.2 vs. 6.9 months	NCT03092674
PD-1	Nivolumab	Nivolumab + low dose cyclophosphamide	R/R AML	II	12	08/2018	01/2022	02/2022	Completed	n.a.	NCT03417154
PD-1	Nivolumab	Nivolumab + azacitidine	childhood R/R AML	I/II	13	11/2019	05/2023	03/2024	Completed	stable disease 33%	NCT03825367
PD-1	Nivolumab	Nivolumab or observation	AML in remission	II	82	07/2025	05/2024	10/2025	Active, not recruiting	n.a.	NCT02275533
PD-1	Pembrolizumab	Pembrolizumab + azacitidine	R/R AML or de novo AML in elderly patients	II	67	07/2016	04/2022	12/2022	Completed	ORR 32%, CR/CRi 14%, median OS 10.8 months	NCT02845297
PD-1	Pembrolizumab	High dose cytarabine followed by pembrolizumab	R/R AML	II	38	08/2016	11/2019	06/2024	Completed	ORR 46%, CR/CRi 38%	NCT02768792
PD-1	Pembrolizumab	Lymphodepletion (fludarabine + melphalan) + autoHSCT followed by pembrolizumab	High-risk AML not eligible for alloHSCT	II	20	09/2016	08/2022	07/2023	Completed	two year OS 68%	NCT02771197
PD-1	Pembrolizumab	Pembrolizumab	AML in remission in elderly patients not eligible for transplantation	II	12	10/2016	06/2020	12/2020	Completed	median OS 43.1 months	NCT02708641
PD-1	Pembrolizumab	Pembrolizumab + decitabine	R/R AML	I/II	10	12/2016	04/2019	04/2019	Completed	ORR 30%, median OS 10 months	NCT02996474
PD-1	Pembrolizumab	Pembrolizumab	Relapsed AML after alloHSCT	Early phase I	12	03/2017	10/2020	11/2020	Completed	ORR 22%, median OS 23.3 months	NCT02981914
PD-1	Pembrolizumab	Pembrolizumab + azacitidine	R/R AML with NPM1-mutation	II	28	08/2019	12/2025	12/2026	Recruiting	n.a.	NCT03769532
PD-1	Pembrolizumab	Pembrolizumab + decitabine or pembrolizumab + decitabine + venetoclax	De novo or R/R AML	I	54	05/2020	11/2025	22/2025	Recruiting	n.a.	NCT03969446
PD-1	Pembrolizumab	Pembrolizumab + azacitidine + venetoclax or azacitidine + venetoclax	De novo AML in unfit patients	II	60	02/2021	06/2023	01/2026	Active, not recruiting	n.a.	NCT04284787
PD-1	Pembrolizumab	Pembrolizumab + cytarabine + idarubicin/daunorubicin or cytarabine + idarubicin/daunorubicin	De novo AML	II	48	02/2021	11/2024	01/2026	Active, not recruiting	n.a.	NCT04214249
PD-1	Camrelizumab	Camrelizumab + decitabine	R/R AML in elderly patients	II	29	04/2020	12/2020	12/2022	Unknown status	n.a.	NCT04353479
PD-1	Tislelizumab	Tislelizumab + venetoclax + HMA	R/R AML	II	67	07/2024	07/2026	07/2027	Recruiting	n.a.	NCT06536959
PD-1 / CTLA-4	Nivolumab / Ipilimumab	Nivolumab + ipilimumab	Relapsed AML after alloHSCT	I	71	05/2013	12/2018	06/2021	Completed	ORR 32%, median OS 56%	NCT01822509
PD-1 / CTLA-4	Nivolumab / Ipilimumab	Nivolumab + azacitidine or nivolumab + azacitidine + ipilimumab or nivolumab + azacitidine + venetoclax	R/R AML or de novo AML in elderly patients	II	150	04/2015	10/2023	10/2023	Completed	ORR 58%, CR/CRi 21%, median OS 9.2 months	NCT02397720
PD-1 / CTLA-4	Nivolumab / Ipilimumab	Nivolumab or ipilimumab, or nivolumab + ipilimumab	Relapsed AML after alloHSCT	I	29	10/2018	12/2024	12/2024	Completed	31% stable disease or remission, median PFS 3.6 months	NCT03600155
PD-1 / TIM-3	Spartalizumab / Sabatolimab	Spartalizumab + decitabine or sabatolimab + decitabine or spartalizumab + sabatolimab + decitabine or sabatolimab or spartalizumab + sabatolimab or sabatolimab + azacitidine	R/R AML or de novo AML not suitable for standard therapy	I	241	07/2017	09/2023	09/2023	Completed	ORR 40%, median PFS 23 months	NCT03066648
PD-1 / LAG3	Nivolumab / Relatlimab	Nivolumab + relatlimab + azacitidine	R/R AML or de novo AML in elderly patients	II	30	05/2021	03/2025	03/2026	Recruiting	n.a.	NCT04913922
PD-L1	Atezolizumab	Atezolizumab + guadecitabine + possibly other immunomodulatory agents	R/R AML or de novo AML in elderly patients	I	40	10/2016	12/2019	12/2019	Completed	n.a.	NCT02892318
PD-L1	Atezolizumab	Atezolizumab + gilteritinib (FLT3 inhibitor)	R/R FLT3-mutated AML	I/II	11	06/2019	05/2021	06/2021	Completed	n.a.	NCT03730012
PD-L1	Durvalumab	Durvalumab + azacitidine or azacitidine	De novo AML in elderly patients	II	213	06/2016	12/2018	12/2021	Completed	ORR 31%, median OS 13 months	NCT02775903
CTLA-4	Ipilimumab	Ipilimumab + DLI	Relapsed AML after alloHSCT	I	21	04/2003	04/2008	04/2008	Completed	median OS 24.7 months	NCT00060372
CTLA-4	Ipilimumab	Ipilimumab	R/R AML	I	42	12/2012	12/2016	12/2016	Completed	median OS 9.8 months	NCT01757639
CTLA-4	Ipilimumab	Ipilimumab + lenalidomide	AML after allo/autoHSCT	II	41	11/2013	05/2024	05/2024	Completed	ORR 70%, CR/CRi 40%	NCT01919619
CTLA-4	Ipilimumab	Ipilimumab + decitabine	R/R AML or de novo AML in elderly patients	I	54	09/2017	10/2022	08/2025	Active, not recruiting	n.a.	NCT02890329
CTLA-4	Ipilimumab	Ipilimumab + CD25/Treg-depleted DLI	Relapsed AML after matched HSCT	I	25	07/2019	05/2024	07/2025	Active, not recruiting	n.a.	NCT03912064
CD70	Cusatuzumab	Cusatuzumab + azacitidine	De novo AML not suitable for intensive chemotherapy	I/II	38	12/2016	08/2022	08/2022	Completed	CR/CRi 50%, median OS 11.5 months	NCT03030612
CD70	Cusatuzumab	Cusatuzumab + azacitidine	De novo AML not suitable for intensive chemotherapy	II	103	07/2019	08/2023	08/2024	Active, not recruiting	n.a.	NCT04023526
CD70	Cusatuzumab	Cusatuzumab + venetoclax or cusatuzumab + venetoclax + azacitidine	De novo AML not suitable for intensive chemotherapy	I	61	12/2019	05/2025	05/2025	Active, not recruiting	n.a.	NCT04150887
CD70	Cusatuzumab	Cusatuzumab + azacitidine	De novo AML not suitable for intensive chemotherapy	I	6	03/2020	07/2021	07/2021	Completed	CR/CRi 16%, 50% stable disease	NCT04241549
CD70	Cusatuzumab	Cusatuzumab + venetoclax + azacitidine or venetoclax + azacitidine	De novo AML not suitable for intensive chemotherapy	II	120	07/2024	01/2027	06/2027	Recruiting	n.a.	NCT06384261
CD70	SEA-CD70	SEA-CD70 or SEA-CD70 + azacitidine	R/R AML or de novo AML	I	140	08/2020	12/2024	11/2026	Recruiting	n.a.	NCT04227847
TIM-3	Sabatolimab	Sabatolimab + venetoclax + azacitidine	De novo AML not suitable for intensive chemotherapy	II	90	09/2020	10/2024	10/2024	Active, not recruiting	n.a.	NCT04150029
TIM-3	Sabatolimab	Sabatolimab or sabatolimab + azacitidine	MRD+ after alloHSCT	I/II	24	09/2021	08/2024	08/2024	Completed	CR/CRi 30%	NCT04623216
TIM-3	TQB2618	TQB2618 + HMA	R/R AML	I	73	04/2022	09/2023	12/2023	Unknown status	n.a.	NCT05426798

Overview of concluded or ongoing clinical trials employing antibodies that target ICs in AML.

*AML* acute myeloid leukemia, *R/R AML* relapsed/refractory acute myeloid leukemia, *CR/CRi* complete remission/complete remission with incomplete hematologic recovery, *OS* overall survival, *ORR* overall response Rate, *HSCT* hematopoietic stem cell transplantation, *alloHSCT* allogeneic hematopoietic stem cell transplantation, *autoHSCT* autologous hematopoietic stem cell transplantation, *DLI* donor lymphocyte infusion, *HMA* hypomethylating agent, *MRD* measurable residual disease, *PFS* progression-free survival.

## Immune checkpoint molecules of the IgSF involved in LSC regulation

### PD-1 (*PDCD1*)

#### Basic cellular biology

PD-1, also known as CD279, is an important immune checkpoint receptor of the CD28-subfamily of the IgSF [14]. Its expression can be induced on immune cells upon activation, and when binding to one of its two ligands (PD-L1 or PD-L2) it dampens the immune response, e.g. by inducing T-cell dysfunction or exhaustion, or by promoting the development of peripherally induced regulatory T-cells [27, 28]. PD-1 signals through an intracellular ITIM and ITSM, which suppress pathways downstream of the TCR and CD28 (e.g., ZAP70, PI3K-AKT, RAS and mTOR) and finally decrease the activation of the transcription factors AP-1, NFAT and NFκB, while increasing the expression of BATF [28, 29]. Apart from its direct role in regulating immune cell activation, PD-1 signaling via SHP-2 has been shown to inhibit myeloid differentiation in melanoma-bearing mice [30].

#### Role in AML and clinical implications

Expression of PD-1 has not only been described on cells of the immune system, but also on certain tumor entities [29, 31, 32]. *PDCD1* mRNA and PD-1 protein expression is higher in CD34^+^ bone marrow cells of patients with myeloid leukemia (CMML and AML) compared to healthy controls [33–35]. Expression of PD-1 on blast cells correlates with a longer disease-free survival [36]. The promoter of *PDCD1* is sensitive to methylation, and PD-1 expression is increased in AML cell lines upon treatment with hypomethylating agents such as decitabine [33, 37]. A poster abstract presented at ASH 2021 showed that in a MLL- and Flt3-driven murine AML model, PD-1 expression on leukemic LSKs was strongly increased. PD-1^+^ AML LSKs had a higher engraftment rate than PD-1^-^ LSKs and induced a more aggressive leukemia with shorter survival. Mechanistically, SHP-1 and phosphorylated (p)-ERK levels were increased in PD-1^+^ compared to PD-1^-^ AML LSKs, a finding that was validated in human PD-1^+^ CD34^+^ AML cells [34]. This suggests that PD-1 signaling in AML LSCs promotes disease progression at least in this molecular defined AML subtype.

### PD-L1 (*PDCD1LG1*)

#### Basic cellular biology

PD-L1, also known as CD274, is one of the two known ligands of PD-1 and a member of the B7-subfamily of the IgSF [14, 16]. Additionally, interactions between CD80 and PD-L1 have been documented [28]. PD-L1 expression has been described on a broad variety of hematopoietic and non-hematopoietic cells and can be constitutive or inducible [27, 38]. In settings of inflammation or infections, PD-L1 expression is induced in hematopoietic, endothelial and epithelial cells to suppress the immune response through binding of PD-1 on activated immune cells [38]. In cancer, gene amplifications and several oncogenic drivers have been reported to directly induce PD-L1 overexpression. PD-L1 is constituted of 290 amino acids, where the intracellular tail is made of only 30 well conserved amino acids lacking conventional signaling motifs [13, 38, 39]. Nevertheless, reverse signaling of PD-L1 has been described in different cancer entities where it was linked to cancer initiation, progression, epithelial-to-mesenchymal transition (EMT) and therapy resistance [13, 40]. PD-L1 was shown to confer protection against pro-apoptotic stimuli within cancer cells even without interacting with PD-1 [39]. Furthermore, PD-L1 reverse signaling is of importance in cancer stem cells (CSCs), where transcription factors like OCT4 or SOX2 were shown to upregulate PD-L1 expression, while PD-L1 signaling activated pathways regulating stemness, such as PI3K/AKT/mTOR, MAPK, or β-catenin [40].

#### Role in AML and clinical implications

In AML, PD-L1 expression on blasts has been described in ca. 20% of adult and a majority of pediatric patients, with higher levels on LSCs compared to AML bulk cells [41–45]. PD-L1 expression on leukemic cells has been associated with poor survival, relapse, and cytogenetic mutations of the adverse risk group [36, 46–48]. *KMT2A* (MLL1), a gene frequently rearranged in AML, directly binds the promoter of PD-L1 to activate its transcription [49]. In addition, MUC1-expressing AML cell lines have a high PD-L1 expression, mediated by MUC1-induced reduction of DICER-expression, a protein crucial for the formation of mature microRNAs. Upon MUC1 suppression, the levels of numerous miRNAs were increased, and miR-34a as well as miR-200c reduced PD-L1 expression [50]. Moreover, inflammatory mediators such as TLR-ligands and IFN-γ increased PD-L1 expression on patient-derived AML blasts [41, 51]. In a murine Asxl1^−/−^xNras^G12D/+^-driven AML model, activated RAS/MEK/ERK signaling induced PD-L1 expression on leukemic blasts [52]. Similar to PD-1, the expression of PD-L1 in AML cell lines is increased upon treatment with hypomethylating agents [33]. Interestingly, in MDS and AML patients, the induction of PD-L1 mRNA expression was associated with absence of clinical response to epigenetic therapy [53].

Functionally, PD-L1 overexpression in AML cell lines stimulated glycolysis and proliferation, and inhibited apoptosis. Rapamycin, an inhibitor of the AKT-mTOR pathway, decreased this PD-L1-induced upregulation of glycolysis-associated proteins [47]. Furthermore, stimulation of PD-L1 reverse signaling in AML cell lines induced gene changes associated with increased fatty acid oxidation and reduced fatty acid synthesis, and stimulated the survival of leukemic cells [54, 55]. Knockdown of PD-L1 in AML cell lines reduced the expression of PI3K and AKT, decreased cell proliferation via G2/M cell cycle arrest and induced cell death [46]. In an MLL-AF9-driven murine AML model, PD-L1 was expressed on Mac-1^+^/c-Kit^+^ LSCs at higher levels than on HSCs, and PD-L1 knockout leukemias were less aggressive in serial transplantations in lethally irradiated and thus immunodeficient mice. While the lack of PD-L1 did not change differentiation or apoptosis, it induced a cell cycle arrest in the G1-phase, concomitantly with a decrease in phosphorylated JNK and Cyclin D2 [56]. Overall, this data indicates that PD-L1 is expressed on AML cells and could, in addition to promoting an immune escape of the leukemic cells, promote tumor progression via metabolic changes, enhanced proliferation and reduced cell death.

### CTLA-4 (*CTLA4*)

#### Basic cellular biology

CTLA-4 is a member of the CD28-subfamily of ICs of the IgSF, and generally opposes the function of CD28 [14, 16]. CTLA-4 on conventional T-cells is upregulated upon activation [57]. A broad evaluation of CTLA-4 expression in different hematopoietic cell types did not show positivity upon scFv-staining of CTLA-4 on the cell surface, but strong positivity in the cytoplasm. Upon stimulation, CTLA-4 was detectable also on the cell surface. For example, CD34^+^ healthy hematopoietic stem cells were negative for CTLA-4 on the surface but showed cytoplasmic staining. GM-CSF stimulation induced CTLA-4 expression at the cell surface [58]. mRNA levels of CTLA-4 were generally low, suggesting pre-synthesis of CTLA-4 and storage in cytoplasmic vesicles [58]. Like CD28, CTLA-4 binds to CD80 and CD86 but with a higher affinity, and thus prevents co-stimulation of immune cells through CD28 [57]. While this mechanism is thought to be the major effector of the immunosuppressive function of CTLA-4, there is an ongoing debate about whether CTLA-4 additionally decreases immune cell activation through signaling via its cytoplasmic tail. In both healthy and cancer cells there is evidence for CTLA-4 cell-intrinsic signaling. For example, CTLA-4 signaling might be involved in regulating differentiation of mesenchymal and neural stem and precursor cells [59]. In different CTLA-4 expressing cancer cell lines, CTLA-4 signaling was able to modulate apoptosis, migration and EMT [59–62].

#### Role in AML and clinical implications

*CTLA4* mRNA levels were increased in PBMCs of AML patients compared to healthy controls, in addition surface CTLA-4 protein was detectable in >50% and cytoplasmic CTLA-4 in nearly 100% of patients [58, 63, 64]. *CTLA4* expression in AML TCGA data correlates with poor prognosis, however *CTLA4* expression did not differ between newly diagnosed and chemoresistant patients [48, 63]. The presence of CT60, a SNP in the 3’ UTR of *CTLA4*, correlates with increased risk of auto-immune diseases and interestingly with increased relapse rates and worse overall survival of AML patients [65]. Stimulation of CTLA-4 through CD80/CD86 induced apoptosis in primary human AML cells [63]. Thus, CTLA-4 may regulate CSCs and its expression in AML correlates with worse overall survival. However, the molecular mechanisms in LSCs have not been defined so far.

### CD86 (*CD86*)

#### Basic cellular biology

CD86, or B7-2, is apart from CD80 the second known ligand for CD28 and belongs to the IgSF B7-subfamily [16]. In contrast to CD80, CD86 is constitutively expressed on APCs and is rapidly upregulated upon stimulation [66]. Furthermore, and contrary to CD80, CD86 reverse signaling in B-cells induced by crosslinking antibodies was able to stimulate their proliferation and the production of IgG1 and IgG2a isotype antibodies [67]. In B-cell lymphoma cell lines, CD86 crosslinking enhanced anti-apoptotic signaling [67].

#### Role in AML and clinical implications

While CD80 is absent on AML cells, CD86 is expressed on approximately 50% of CD45^+^CD34^+^c-Kit^+^ cells [63, 68–70]. Similarly to CD80, CD86 expression is increased on primary AML samples by Ara-C exposure [71]. Additionally, CD86 but not CD80 expression is increased on AML cell lines and primary samples upon histone deacetylase inhibitor treatment. Interestingly, this does not seem to be uniquely a result of differentiation, as ATRA-stimulated differentiation of AML cell lines did not lead to an upregulation of CD86 [72]. LSD1/KDM1A inhibition induced the differentiation markers CD11b and CD86 in MLL-rearranged AML resulting in decreased clonogenicity and proliferation [73]. In TCGA data, high CD86 expression was positively correlated with gene signatures of immune cells, as well as with a worse prognosis of AML patients [74]. Similarly, CD86-positivity of mononuclear cells of newly diagnosed AML patients is associated with significantly worse survival [68, 69]. AML patient samples cultured in vitro for several weeks lost their progenitor surface markers CD33, CD13 and CD34, while increasing the expression of CD80 and CD86 [75]. To summarize, there seems to be an inverse correlation between progenitor markers and CD86 expression, however it remains to be determined whether CD86 expression is functionally involved in LSC regulation and differentiation.

### CD276 (*CD276*)

#### Basic cellular biology

CD276, or B7-H3, is yet another member of the B7-subfamily of IgSF ICs [16]. Although it is broadly expressed on mRNA level in most tissues, protein cell surface expression is limited to some immune cells [76]. While its binding partners are unknown, CD276-mediated co-stimulatory as well as co-inhibitory effects on the immune system have been described [76]. CD276 is frequently overexpressed in various cancer types, such as breast, pancreatic, gastric, lung, and prostate cancer, as well as melanoma [76, 77]. High CD276 has been associated with better prognosis in gastric and pancreatic cancer, while in NSCLC it was a poor prognostic marker [77]. CD276 promoted progression, migration, drug resistance and stemness, possibly via the JAK/STAT3 pathway, in different solid cancer cell lines [77]. In glioblastoma, CD276 expression correlated with CSC-related genes and *MYC*, and CD276 was suggested as an inhibitor of the TGF-β pathway and differentiation of CSCs [78].

#### Role in AML and clinical implications

In several public datasets, *CD276* mRNA expression in AML patient samples was higher compared to healthy controls, and high expression correlated with worse survival [76]. CD276 was detected on CD45^+^CD34^+^c-Kit^+^ cells in 30% of AML patient samples, while it was absent in healthy donors [70]. Additionally, *CD276* mRNA was higher in LSCs compared to normal HSCs, and CD34^+^ cells of acute myeloid and lymphoid leukemia patients had higher CD276 protein expression than their CD34^-^ counterparts [76, 79]. In contrast, while Guery et al. confirmed that 30% of all AML patients expressed CD276, they did not find an increased expression in CD34^+^CD38^-^ cells and documented a significantly improved event-free survival in patients with CD276^+^ AML [80]. Nevertheless, in patients from the TCGA dataset with high *CD276* mRNA, several oncogenic gene signatures were enriched, while tumor suppressors were deleted [76]. The expression of two genes associated with EMT (*TWIST1* and *MMP7*) was significantly associated with CD276 expression [76]. CD276 is expressed as two isoforms, 2Ig (membrane and soluble form) and 4Ig (potentially only membrane-bound form). 4Ig is the main isoform expressed in most malignancies. However, in AML cell lines and patient samples, the 2Ig isoform expression was increased compared to healthy controls and correlated with a worse overall survival, while 4Ig expression did not [81]. In the AML cell line U937, CD276 knockdown decreased survival, proliferation, colony formation, and tumor-growth in a xenograft model. Upon CD276 knockdown, cells also showed reduced migration and invasion capacity in a transwell assay, which was associated with decreased expression of *MMP2* and *MMP9* mRNA. Furthermore, U937 cells with reduced CD276 were more sensitive to chemotherapy [82]. In summary, expression of CD276 has been detected on a significant portion of primary AML samples and a functional role in AML and LSCs has been documented, but correlations of CD276 expression to clinical outcomes varied. This, however, could also be attributed to different detection methods and different isoforms being assessed.

### VTCN1 (*VTCN1*)

#### Basic cellular biology

VTCN1, also known as B7-H4, B7S1, or B7x, is the 7^th^ member of the B7-subfamily of immune checkpoints of the IgSF [16]. While VTCN1 mRNA is detected in a variety of tissues, its protein expression is restricted to epithelial and some non-epithelial tissues such as bone marrow-derived mesenchymal stem cells [83]. Its expression on cells of the hematopoietic system is unclear [83]. Functionally, VTCN1 exerts an inhibitory effect on the immune system, even though its binding partners are yet to be determined [83]. Inflammatory cytokines that drive the expression of other B7-family members do not lead to the upregulation of VTCN1 on cancer cells, while immunosuppressive cytokines such as TGF-β1 and IL-10 do [83]. Furthermore, a hypoxic environment was able to increase VTCN1 expression in human cancer cells [83]. VTCN1 overexpression is found frequently in solid tumors, such as in breast, kidney, ovary, prostate, stomach, skin, pancreas, brain, liver, and lung cancer [83]. In most cases, high levels of VTCN1 are associated with worse prognosis [83]. Interestingly, VTCN1 not only exists as membrane bound, but also as soluble form [83]. Human VTCN1 contains a nuclear localization signal, and its translocation into the nucleus of renal cell carcinoma cells was necessary for the proliferation enhancing effect of the molecule [84]. Furthermore, in colorectal cancer, VTCN1 correlated with worse survival rates, and a knockdown inhibited spheroid formation, cell migration and invasion [85]. In esophageal squamous cell carcinoma, VTCN1 expression was associated with the expression of cancer stemness proteins (SOX9, LSD1, OCT4, LGR5) and an activated PI3K/AKT/NFκB pathway [86].

#### Role in AML and clinical implications

In AML patients, VTCN1 expression levels correlated with better overall survival [87]. Human cord blood CD34^+^ cells transduced with the MLL-AF9 oncogene showed a strong upregulation of VTCN1 compared to non-transduced counterparts [88]. In a murine MLL-AF9-driven AML model, leukemic cells derived from VTCN1^−/−^ mice were more aggressive in serial transplantations, and LSC frequency was 10-fold increased [87]. VTCN1^−/−^-LSCs showed reduced capacity to differentiate, and RNAseq revealed that VTCN1^−/−^-LSCs expressed increased levels of genes important for CSCs (*Rcor2*, *Klf2*, *Klf4*, *Klf6*, *Hif1a*) [87]. *Rcor2* downregulation, and thus a reduced *Runx1* level, was shown to be the main mediator of how VTCN1 inhibited leukemogenesis, potentially through interacting with PTEN and AKT [87]. Thus, the effect of VTCN1 in the regulation of solid tumors and leukemia seems different: VCTN1 propagates CSCs in solid tumors but reduces LSCs in AML.

### VISTA (*VSIR*)

#### Basic cellular biology

VISTA, known as well as B7-H5 or PD-1H, is a member of the IgSF subfamily CD28 [14]. It is broadly expressed on hematopoietic cells, and can deliver inhibitory signals to T-cells by functioning both as ligand and as receptor [89]. VISTA has been described to interact with VISTA, VSIG-3, Galectin-9, and - in a pH-dependent manner - PSGL-1 [89, 90]. VISTA expression has been described on melanoma and NSCLC cells [89]. In gastric adenocarcinoma metastases, patients with a more mesenchymal-like cancer subtype had high expression of VISTA [91].

#### Role in AML and clinical implications

In the TCGA database, *VSIR* mRNA levels were higher in AML patients compared to all 30 other human cancer types, and VISTA protein is detectable in most AML samples [89]. Consistent with its expression on normal myeloid cells, *VSIR* mRNA levels were highest in the M4 (myelomonocytic) and M5 (monocytic) FAB subtypes [89]. Furthermore, CD34^+^ cells derived from BM of AML patients showed a higher VISTA expression than CD34^-^ cells [92]. Expression of VISTA on AML cells is regulated by STAT3 signaling [92]. *VSIR* mRNA levels are a negative prognostic factor in AML [89]. Yasinska et al. showed that VISTA specifically interacts with Galectin-9, a molecule better known for its role as ligand for TIM-3, and involved in the TIM-3/Gal-9 autocrine loop that stimulates LSC self-renewal [90, 93]. Soluble VISTA and Galectin-9 levels were elevated in the serum of AML patients compared to healthy controls [90]. An abstract presented at ASH 2023 showed a reduction of AML cell line growth in NSG mice when treated with anti-VISTA antibody [94]. However, upon overexpression of VISTA in a murine syngeneic AML cell line model, tumor progression did not differ compared to control cells when transplanted into NSG mice [89].

### CD28H (*TMIGD2*)

#### Basic cellular biology

CD28H has recently been identified as the co-stimulatory receptor for HHLA2 belonging to the CD28 subfamily of IgSF ICs. Alternative names for CD28H are TMIGD2 or IGPR-1 [95]. CD28H is expressed on naïve T-cells, NK cells, ILCs, pDCs, and tissue resident T-cells [96]. HHLA2 and CD28H do not have orthologs in mice [97]. In colon cancer, CD28H was upregulated and promoted multicellular aggregation, tumor growth and resistance to chemotherapy [98].

#### Role in AML and clinical implications

72.1% of AML patients express CD28H on CD34^+^ cells [97]. CD28H protein expression was higher in CD34^+^ AML cells compared to healthy CD34^+^ controls [97]. In the TCGA dataset, high *TMIGD2* expression correlated with worse survival [97]. RFX1 has been identified as a transcription factor driving *TMIGD2* expression [97]. Interestingly, CD28H protein expression was specifically elevated in CD45^dim^SSC^low^Lin^-^CD34^+^CD38^-^ cells, a subpopulation that is highly enriched for LSCs [97]. Treatment of different AML cell lines with a differentiation-inducing agent led to decreased CD28H expression [97]. Upon shRNA-mediated knockdown of CD28H, AML cell lines and primary AML samples showed decreased proliferation, survival and colony formation, concomitant with increased differentiation. Furthermore, levels of phosphorylated SHP-1, ERK and CREB were reduced, and CREB overexpression could partially rescue the phenotype [97]. Interestingly, knockdown of the ligand HHLA2 did not result in functional changes. Further investigation revealed that CD28H on leukemic cells formed cis-homodimers, which could be blocked by anti-CD28H antibodies [97]. Thus, CD28H directly regulates LSC stemness and disease progression.

### TIM-3 (*HAVCR2*)

#### Basic cellular biology

TIM-3 belongs to the TIM subfamily of immune checkpoints of the IgSF and was originally identified on IFN-γ-producing CD4^+^ and CD8^+^ T-cells, where it functions as an inhibitory receptor [99, 100]. Since then, many other immune cells have been described to express TIM-3, such as Tregs, macrophages, NK cells, and DCs [99]. TIM-3 is now suggested to be a marker of exhausted and dysfunctional T-cells and to mediate immune suppression in both the adaptive and the innate immune system [99]. The transcription factors T-bet, NFIL3, PRDM1 and MAF were found to control TIM-3 expression [100]. Several ligands have been described to interact with different regions of TIM-3, including Galectin-9, Phosphatidylserine, CEACAM1 and HMGB1 [100]. TIM-3 does not contain any known inhibitory signaling motifs in its intracellular tail, and it is hypothesized that signaling in T-cells functions via conserved tyrosine residues which interact with BAT3 and FYN [100]. Interestingly, TIM-3 was also found to be expressed on liver cancer cells, where it promoted tumor progression via NFκB/IL-6/STAT-3 pathway, and cervical cancer, where TIM-3 expression was associated with shorter overall survival and advanced cancer stages, and promoted migration and invasion of cancer cells [101, 102].

#### Role in AML and clinical implications

TIM-3 is expressed on LSCs in the majority of AML patients (78.5%), while its expression decreases on more differentiated blasts and is absent on healthy HSCs [99, 103, 104]. Accordingly, TIM-3^+^ AML cells have higher engraftment rates in secondary recipient mice than TIM-3^-^ AML cells [104]. TIM-3 expression on blasts is associated with poor prognostic markers and shorter overall survival [105, 106]. Kikushige et al. describe an autocrine loop on LSCs between TIM-3 and its ligand Galectin-9, which is presumably involved in leukemic transformation and essential for LSC self-renewal and stemness regulation [93]. Upon Galectin-9 ligation to TIM-3, phosphorylation levels of ERK, AKT and NFκB, and nucleic levels of β-catenin were increased, suggesting an activation of these pathways known to play important roles in LSC regulation [93, 99]. Blocking this autocrine loop with neutralizing anti-Galectin-9-antibodies reduced leukemic engraftment, while it did not affect normal hematopoiesis [93]. A phase 1b trial with sabatolimab (NCT03066648), a TIM-3 monoclonal antibody, in combination with hypomethylating agents showed an overall response rate of 40.0% among 40 AML patients [107]. In patients with residual disease after allogeneic hematopoietic stem cell transplantation, sabatolimab with or without combination of azacitidine is currently being tested in a phase 1/2 study (NCT04623216). Furthermore, a phase 2 study of sabatolimab in combination with azacitidine and venetoclax (NCT04150029) in first-line unfit patients is ongoing. TIM-3 is therefore a promising target in AML. Blocking TIM-3 may activate leukemia-specific immune responses and directly inhibit LSCs expansion.

### LAIR-1 (LAIR1)

#### Basic cellular biology

LAIR-1, also known as CD305, is a member of the LAIR-subfamily of IgSF ICs [14]. It binds to collagen and collagen-domain containing proteins such as surfactant protein D and C1q [108]. LAIR-1 is expressed on most cells of the immune system and generally exerts an inhibitory effect in vitro, mediated by two ITIMs on its intracellular domain [108]. In ovarian cancer, LAIR-1 knockdown increased cell proliferation, migration, invasion and colony formation, and in cervical cancer, LAIR-1 overexpression suppressed cell proliferation and survival. In addition, in hepatocellular carcinoma, LAIR-1 expression is associated with the grade of the disease [108].

#### Role in AML and clinical implications

LAIR-1 is highly expressed in human AML patients and AML cell lines [109, 110]. However, data on its role in AML is conflicting. Early studies reported a leukemia-suppressing effect of LAIR-1 on AML cells, where LAIR-1 signaling induced apoptosis and reduced proliferation via SHP-1 and inhibition of NFκB translocation into the nucleus [111, 112]. Recent data supports this, as Lovewell et al. show increased apoptosis and decreased colony formation and xenograft growth in AML patient samples upon treatment with an agonistic antibody against LAIR-1. LAIR-1-agonistic antibody treatment induced SHP-1 phosphorylation and decreased the activation of AKT, mTOR and NFκB [110]. However, conflicting data came from another group documenting that the viability of AML cells and LSCs depends on LAIR-1 expression: LAIR-1 knockdown strongly enhanced AML cell line apoptosis and inhibited xenograft growth, and Lair-1-deficient MLL-AF9 and AML1-ETO9a induced murine AML models had a reduced LSC frequency and a longer survival. These effects appeared to be mediated through a phosphatase-independent function of SHP-1 recruiting CAMK1 to LAIR-1 [109]. Thus, LAIR-1 has opposite effects on AML cells and LSCs, dependent on the model analyzed.

## Immune checkpoint molecules of the TNFRSF/TNFSF involved in LSC regulation

### TNFRSF3 *(LTBR)*

#### Basic cellular biology

LTβR, or TNFRSF3, belongs to the TNFRSF of ICs and is mostly known for its role in orchestrating lymphoid organogenesis and inflammation. It is expressed on endothelial cells, stromal cells, dendritic cells and macrophages, but absent on lymphoid cells. Meanwhile, lymphoid cells predominantly express its ligands LTαβ and LIGHT. LTβR signaling is essential for the development and maintenance of secondary lymphoid organs and for the homeostasis of immune cells such as NK cells and neutrophils. Via NFκB activation, LTβR signaling induces proinflammatory cytokines [113]. In multiple solid tumor cell lines, activation of LTβR signaling induced cell death and inhibited tumor growth [114].

#### Role in AML and clinical implications

In healthy HSCs and CML LSCs, LTβR/LIGHT signaling maintained the pool of stem cells by inhibition of cell cycling and favoring symmetrical over asymmetrical cell division [115]. In AML patients with RUNX-translocations, *LTBR* mRNA was found to be elevated in CD34^+^CD117^hi^-G2M cells, while being absent in other AML and healthy cell subsets [116]. An abstract presented at ASH 2023 showed that AML LSCs express both LTβR and LIGHT, while LIGHT was absent on healthy HSCs. Low levels of *TNFSF14* (LIGHT) or *LTBR* mRNA correlated with improved survival. Inhibition of LTβR/LIGHT signaling through KO of the receptor or ligand, or addition of biologically inactive ligand reduced colony formation and LSC frequency, while it prolonged survival in a murine MLL-AF9-driven AML model. Similarly, siRNA-mediated knockdown of LIGHT reduced colony formation of patient samples. A LIGHT-blocking antibody was able to reduce colony formation and stemness signature genes in LSCs and enhanced differentiation, while HSPCs of healthy controls were not affected [117]. Thus, targeting LSCs through blockade of LIGHT and thus inhibiting LTβR/LIGHT signaling is a promising therapeutic approach in AML.

### CD27 *(CD27)* and CD70 *(CD70)*

#### Basic cellular biology

CD27, or TNFRSF7, is a receptor of the TNF superfamily of ICs. It is expressed on naïve T- and memory B- and T-cells, Tregs, and on a subset of NK cells [118]. Interactions of CD27 with its only ligand, CD70, deliver costimulatory signals to CD27-expressing cells, which activate NFκB and c-JUN pathways, finally enhancing cell proliferation, survival and differentiation [118]. CD70, otherwise known as TNFSF7 or CD27L, also belongs to the TNF superfamily of ICs, and is physiologically only transiently expressed on activated B- and T-cells, NK cells and mature DCs [118]. CD70 reverse signaling has been described on B-, T- and NK-cells, stimulating cell proliferation and activation via PI3K/AKT and MEK signaling pathways [119–121]. Apart from its expression on immune cells, murine - but not human - HSCs and progenitor cells express CD27, and stimulation with CD70 inhibited colony formation and leukocyte differentiation, suggesting a negative feedback system [118, 122]. While CD27 expression has not been reported for solid tumors, CD70 was found to be overexpressed and associated with worse prognosis on various tumor entities [118]. Metastatic tumors showed an upregulation of CD70 expression compared to primary tumors, and CD70 seems to be involved in CSC and EMT pathways [118]. In B- and T-cell malignancies, CD70 with or without co-expression of CD27 has been described, and its expression is associated with unfavorable prognosis [118].

#### Role in AML and clinical implications

In CML patients and a murine CML model, LSCs were found to express CD27 which stimulated colony formation, proliferation and cell cycle progression via TRAF2/TNIK-mediated Wnt/β-catenin-pathway activation [123]. Inhibition of the BCR/ABL tyrosine kinase increased CD70 expression in CML LSCs, thus enhancing their drug resistance through activation of the Wnt/β-catenin-pathway [124]. CD27 protein was detected on 96% and CD70 protein on 100% of AML samples while it was absent from healthy HSPCs [125]. Soluble CD27, indicative of active signaling, was abundantly detected in serum of AML patients and correlated with worse prognosis [125]. Blockage of CD27/CD70 signaling reduced stem cell gene signature pathways such as Wnt, JAK/STAT, Hedgehog and TGF-β-signaling, resulting in reduced proliferation and self-renewal, while enhancing differentiation in primary AML cells [125]. Based on this preclinical data, a dose-escalation phase 1 trial (NCT03030612) with the ADCC-optimized CD70-targeting antibody cusatuzumab in combination with azacitidine was performed. The overall response rate in the phase 1 dose-escalation part was 100%. Four out of nine evaluable patients (44%) with CR/CRi achieved minimal residual disease (MRD)-negativity [126]. Notably, cusatuzumab treatment significantly reduced LSCs and activated gene signatures related to myeloid differentiation and apoptosis [126]. The phase 2 extension phase (NCT03030612) revealed an objective response (≥partial remission) in 19/38 patients, and 14/38 achieved complete remission [127]. The combination of cusatuzumab with azacitidine and venetoclax was tested in the ELEVATE trial (NCT04150887) and resulted in a CR rate of 77.3% (including CRi) [128]. The combination is now tested in a prospective randomized trial (NCT06384261). Thus, targeting CD70 might become the first IC that eliminates LSCs in AML patients.

### TNFRSF17 (TNFRSF17)

#### Basic cellular biology

The 17^th^ member of the TNF receptor superfamily, TNFRSF17, also known as B-cell maturation antigen (BCMA) or CD269, is an IC receptor binding two ligands, TNFSF13 (APRIL) and TNFSF13B (BAFF) [129]. While BCMA expression has been reported on circulating monocytes, B-, T- and NK-cells, as well as on CD34^+^ HSPCs, this was not confirmed in other studies, a difference that could be attributed to differential sample preparation methods inducing cell activation [129]. As the name suggests, its role is most extensively studied in B-cell maturation and differentiation, where it promotes survival, maturation and differentiation [129]. Signaling pathways induced by ligand-binding to BCMA were shown to vary depending on the cellular context, and include the activation of the NFκB pathway which in some cases induced c-MYC, and JNK-kinases, as well as inhibition of apoptosis through downregulation of pro-apoptotic proteins (e.g. BIM, BAX) and upregulation of anti-apoptotic proteins (e.g. BCL-2, BCL-xL) [129]. BCMA protein has been detected on hepatocellular carcinoma, squamous cell carcinoma and glioma. In all three cancer subtypes, at least one of its ligands was also upregulated [129]. In hepatocellular carcinoma, APRIL binding to BCMA induced cell cycle arrest, while in glioma, high BCMA correlated with a higher tumor grade [130, 131]. In several hematological malignancies, especially in multiple myeloma and moderately in ALL, BCMA expression on malignant cells has been reported. In multiple myeloma, elevated levels of its soluble form were an independent prognostic marker for poor clinical outcomes [132].

#### Role in AML and clinical implications

BCMA has been detected on a subset of blasts in AML patients. Interestingly, patients who achieved complete remission after induction chemotherapy had a higher frequency of BCMA^+^CD33^+^ blasts at baseline, compared to non-responders. In contrast, non-responders had increased levels of APRIL^+^CD33^+^ and BAFF^+^CD33^+^ blasts at baseline [133]. In a murine model of MLL-AF9, APRIL was found to increase the leukemia-initiating capacity. APRIL-stimulation of leukemic cells reduced apoptosis and induced cell cycling. Interestingly, healthy myeloid BM cells but not leukemic cells secrete APRIL, indicating an involvement of the TME. Of the two known receptors for APRIL, only BCMA was expressed on leukemic cells, suggesting that the APRIL/BCMA interaction expands murine AML cells [134]. In accordance with these findings, APRIL has been found to have anti-apoptotic and chemoresistance-enhancing effects in human AML [133, 135].

## Clinical implications

Blocking ICs has become a standard of care in many solid tumors. The PD-1/PD-L1 signaling pathway is crucial for the induction of T-cell dysfunction in tumors and many PD-1 or PD-L1 blocking antibodies are approved in different indications. In addition, blocking CTLA-4 antibodies are used mainly in combination with PD-1/PD-L1 blocking antibodies in melanoma, lung carcinoma and mesothelioma. A role of the PD-1/PD-L1 pathway in immune evasion has been documented in several murine leukemia models. However, clinical trials with PD-1/PD-L1 blocking antibodies as monotherapy had disappointingly low response rates [25]. Several studies with different PD-1/PD-L1 blocking antibodies are ongoing, either at a timepoint when most of the leukemia bulk has been eliminated by prior chemotherapy, in combination with chemotherapy or in combination with hypomethylating agents that upregulate the expression of PD-L1 and antigen presentation on leukemia cells. However, T-cell dysfunction may be more severe in the BM of AML patients than in solid tumors and less dependent on a single ICs such as PD-1/PD-L1 [8, 9, 26]. Accordingly, combinations of IC blockades are being tested in AML [24]. Furthermore, PD-L1-targeting antibodies may induce reverse signaling and promote the survival and proliferation of LSCs.

LSCs are the main drivers of relapse in AML, and their elimination is therefore crucial to achieve long-term remission and cure [1]. Besides activating the immune system, targeting defined ICs in AML might directly eliminate leukemic cells and especially LSCs. Clinical studies with TIM-3 blocking antibodies in AML and MDS in combination with azacitidine and venetoclax are ongoing (NCT04623216, NCT04150029). Similarly, a randomized trial testing the CD70-targeting antibody cusatuzumab in combination with azacitidine and venetoclax (NCT04150887) is currently recruiting in the US and in Europe. So far, few ICs with a defined function on AML LSCs have been identified. However, as summarized above and in Tables 1 and 2, many ICs are expressed by AML LSCs and their expression usually correlates with worse overall survival. Thus, even if an IC does not have a direct functional role on LSCs, targeting these cell surface molecules might eliminate LSCs. For example, the CD70-targeting antibody cusatuzumab has an ADCC optimized Fc-part that recruits effector cells such as NK cells. In preclinical models, the ADCC-enhanced antibody eliminated LSCs more efficiently than blocking-only antibodies [126]. Moreover, ICs might be targeted by bi-specific antibodies recruiting CD3^+^ T-cells or by specific CAR-T cells. For example, different CD70 CAR-T cell constructs have been generated and tested in preclinical models and two CD70 CAR-T cells studies are currently recruiting (NCT04662294, NCT06492304) [136–138].

Thus, although the classical IC blockade of the PD-1/PD-L1 signaling in AML is not as efficacious as in solid tumors, many other IC molecules have been identified on AML blasts and LSCs with the potential to become therapeutic targets in the future.
